# Potential Health Consequences of Regular Blood Donation: Current Evidence and Remaining Uncertainties

**DOI:** 10.3390/jcm15176847

**Published:** 2026-09-04

**Authors:** Sarah Berli, Mara Sofie Kaiser, Eméry Schindler, Dimitrios A. Tsakiris

**Affiliations:** Blood Transfusion Service SRC Zurich, Swiss Red Cross, CH-8952 Schlieren, Switzerland; sarah.berli@uzh.ch (S.B.); mara-kaiser@live.de (M.S.K.); e.schindler@zhbsd.ch (E.S.)

**Keywords:** blood donation, donor health, iron deficiency, clonal hematopoiesis CHIP, lymphopenia, plasmapheresis, plateletpheresis, immunoglobulin, vitamin deficiency, hemovigilance, ferritin, apheresis

## Abstract

Blood donation sustains an estimated 118 million annual donations worldwide, and while advances in infectious disease screening have greatly reduced recipient risk, the long-term health of repeat donors has received comparatively less attention. This cumulative review synthesises current evidence on the principal acquired complications of regular blood donation across all modalities—whole blood, plasmapheresis, and plateletpheresis—addressing iron deficiency, plasma protein depletion, apheresis-related lymphopenia, clonal haematopoiesis of indeterminate potential (CHIP), and micronutrient deficiency. Iron deficiency affects 15–36% of frequent whole blood donors and is systematically underdetected by haemoglobin-based screening alone; ferritin-guided interval adjustment and oral supplementation are effective countermeasures. Regular plasmapheresis depletes immunoglobulins (IgG below normal in 5–15% of high-frequency donors), albumin, and coagulation factors, compounded by cumulative citrate-mediated hypocalcaemia. Plateletpheresis causes measurable subset-selective T-cell and NK-cell depletion, with CD4^+^ counts below clinically significant thresholds in a minority of the most frequent donors, an observation of unknown significance. Emerging molecular data indicate that repeated haematopoietic stress selectively enriches low-risk, EPO-responsive DNMT3A clonal variants without increasing malignant risk. Regular blood donation if properly applied is a safe process. Across all domains, current eligibility criteria based on haemoglobin alone turn out to be inadequate; a shift to multiparameter, individualised and targeted donor monitoring—integrating ferritin, serum proteins, differential leucocyte counts, and micronutrient profiling—is both evidence-suggested and ethically worth considering to sustain the long-term health of voluntary donor populations.

## 1. Introduction

The voluntary, non-remunerated donation of blood and blood components forms the ethical and operational foundation of modern transfusion medicine. The World Health Organisation endorses voluntary, unpaid donation as the safest source of blood, and most high-income countries have structured their supply systems accordingly. Despite the central importance of donor populations to healthcare delivery, the long-term health of repeat donors has historically received comparatively less scientific scrutiny. The implicit assumption underlying many regulatory frameworks—that donation, within approved frequency limits, is physiologically inconsequential to the donor—is increasingly challenged by a growing body of laboratory, epidemiological, and molecular evidence.

Donation modalities vary substantially in their biological impact profiles. Whole blood donation imposes primarily a loss of cellular and plasma components simultaneously, with iron depletion as the dominant chronic consequence. Plasmapheresis selectively removes protein-rich plasma while returning cellular elements, exposing high-frequency donors to progressive depletion of immunoglobulins, albumin, and coagulation factors, as well as cumulative citrate effects. Plateletpheresis, while preserving erythrocytes and plasma, inadvertently co-collects lymphocytes during centrifugal separation, with possible consequences for cellular immune competence in regular donors. Across all modalities, the bone marrow responds to repeated haematopoietic stress with increased stem cell proliferative activity, a process now understood to have implications for the dynamics of clonal haematopoiesis.

This review consolidates this knowledge into a unified narrative examining the principal acquired health consequences of regular blood donation. It addresses, in sequence: iron deficiency associated with whole blood donation; the distinctive complications of regular plasma donation by apheresis; acquired absolute lymphopenia and immune perturbation in platelet apheresis donors; the biology and clinical implications of clonal haematopoiesis of indeterminate potential (CHIP) in the context of regular donation; and the emerging evidence on micronutrient—particularly vitamin—deficiencies in regular donors. For each complication domain, we describe pathogenesis, epidemiology, clinical presentation, and evidence-based donor protection strategies. We conclude with an integrated perspective on the reform of donor health monitoring frameworks and the future research agenda required to protect the millions of individuals who sustain blood supply systems worldwide.

Concerning the literature search methodology PubMed/MEDLINE, Web of Science and Scopus were searched as databases and studies published from 2000 to current were considered. The search strategy included combinations of terms related to blood donation, blood donors, iron status, haematological parameters, physiological effects, etc. Reference lists of relevant articles were also screened to identify additional publications. Articles were selected based on their relevance to the objectives and scope of the narrative review. Original research articles, systematic reviews, meta-analyses, clinical guidelines, and/or other relevant publications were considered. Because this manuscript was designed as a narrative review, the literature search was intended to identify and synthesise relevant evidence rather than to provide an exhaustive systematic review of all available studies.

## 2. Iron Deficiency as an Acquired Complication of Regular Blood Donation

### 2.1. Pathogenesis

Iron deficiency is the most prevalent acquired micronutrient complication attributable to regular blood donation, arising from the straightforward biophysical fact that each whole blood donation of 450–500 mL removes approximately 200–250 mg of elemental iron—predominantly as haemoglobin within donated erythrocytes, with additional contributions from plasma transferrin and ferritin [1,2,3]. This single-donation loss represents the equivalent of three to four months of dietary iron absorption at normal efficiency. The principal homeostatic regulator of systemic iron balance, hepcidin—a hepatic peptide hormone that suppresses the iron exporter ferroportin on enterocytes and macrophages—falls sharply following donation, upregulating intestinal absorption as a compensatory response [4]. In practice, however, this compensatory absorption rarely fully offsets donation-related losses, because the bioavailability of dietary non-haem iron—the dominant dietary iron form in Western populations—ranges from only 2–8%, far below the theoretical absorptive ceiling of 4–5 mg per day.

In donors whose inter-donation intervals are shorter than the three to four months required for complete iron store restoration, each successive donation initiates a stepwise reduction in ferritin. The sequential trajectory proceeds from initial iron store depletion (falling ferritin, stable haemoglobin) through iron-deficient erythropoiesis (rising serum transferrin receptor, falling transferrin saturation, hypochromic microcytic reticulocytes) to frank iron deficiency anaemia [1]. Critically, haemoglobin may remain above donation thresholds throughout much of this trajectory, rendering standard haemoglobin-based eligibility screening structurally incapable of detecting the complication at its earliest and most correctable stage.

### 2.2. Incidence and Epidemiology

The epidemiological evidence for donation-related iron deficiency is internationally consistent and substantial. In the landmark REDS-II RISE study—a large prospective cohort of 2425 American donors—36% of frequent female donors and 23% of frequent male donors had ferritin below 12 µg/L, despite acceptable haemoglobin at donation [3]. The UK INTERVAL trial, randomising 45,000 donors to different inter-donation intervals, demonstrated that male donors at 8-week intervals had ferritin decrements approximately 2.5-fold greater than those donating at 12-week intervals, without a proportionate increase in haemoglobin deferral, confirming the dissociation between haemoglobin and iron store status in this population [5,6]. Cross-sectional surveys across Europe, North America, and Australia consistently document ferritin below 15 µg/L in 14–30% of regular female donors and 8–12% of regular male donors, compared with 5–8% and 2–3%, respectively, in age-matched non-donors [2,7].

Risk is not uniformly distributed. Premenopausal women bear a disproportionate burden from the additive effect of menstrual iron loss and donation-related depletion; studies report ferritin below 15 µg/L in 25–40% of this subgroup. Donors following plant-based dietary patterns face amplified risk due to exclusive reliance on non-haem iron. First-time donors with pre-existing subclinical iron depletion—common among young women and adolescents—may develop measurable deficiency after as few as one or two donations. The recently published FIND’EM trial, a stepped-wedge cluster-randomised study of 412,888 Dutch donors, demonstrated that nationwide implementation of ferritin-guided donation intervals significantly reduced the prevalence of iron deficiency compared to interval-fixed management [8].

### 2.3. Clinical Presentation

The clinical manifestations of donation-related iron deficiency span a spectrum from entirely asymptomatic iron store depletion, detectable only by laboratory measurement, to the full syndrome of iron deficiency anaemia. In the pre-anaemic phase, non-specific symptoms emerge that are frequently attributed to unrelated causes: fatigue, reduced physical endurance, and cognitive impairment—including poor concentration and impaired short-term memory—reflecting iron’s role in dopaminergic neurotransmission and cerebral myelination, effects that may be present at ferritin levels well above the anaemia threshold [2,5,9]. Restless legs syndrome has been specifically linked to low ferritin in blood donor studies, with evidence of a threshold effect below ferritin 50 µg/L. In donors with frank iron deficiency anaemia, the classical haematological syndrome appears with hypochromic microcytic erythrocytes, pallor, exertional dyspnoea, brittle nails, glossitis, and angular cheilitis.

### 2.4. Donor Protection Strategies

A multicomponent evidence-based approach to protection against donation-related iron deficiency has emerged from prospective trials. Ferritin-based eligibility screening—replacing or supplementing haemoglobin measurement—enables detection of iron depletion before anaemia supervenes; thresholds of 15–20 µg/L are employed by progressive blood services in Denmark, the Netherlands, and the United Kingdom [8]. Extending minimum inter-donation intervals—to 12 weeks for men and 16 weeks for women in some jurisdictions—provides additional time for iron store recovery; dynamic, ferritin-guided intervals represent the most precise refinement of this approach [8,10,11]. Short-course oral iron supplementation following donation—ferrous sulphate 325 mg (65 mg elemental iron) daily for 60 days—has been demonstrated in the HEIRS study and RISE trial to significantly attenuate ferritin decline, reduce subsequent deferral rates, and improve quality of life [12]. Structured dietary counselling should emphasise iron-rich food sources, vitamin C co-ingestion to enhance non-haem iron absorption, and the inhibitory effects of polyphenols, phytates, and calcium on absorption. Transparent communication of ferritin results to donors—framed within clear educational messaging about iron physiology—is both an ethical obligation and a practical behaviour-change intervention [13].

## 3. Acquired Complications of Regular Plasma Donation by Apheresis

### 3.1. Overview and Context

Plasmapheresis—the selective collection of plasma by automated apheresis technology with reinfusion of cellular components—is an indispensable pillar of the global supply of therapeutic plasma proteins, including intravenous immunoglobulins, albumin, and coagulation factor concentrates. Unlike whole blood donation, plasmapheresis retains erythrocytes, leucocytes, and platelets, allowing a higher permissible donation frequency: regulatory frameworks in the United States permit up to 104 sessions per year under current FDA guidelines, a frequency substantially exceeding the World Health Organisation recommendation of 25–30 non-remunerated donations annually [14]. This apparent selectivity has historically been cited to justify intensive donation schedules; however, the assumption that plasma removal is physiologically inconsequential beyond its acute volume effects is increasingly untenable in light of the accumulating evidence of progressive depletion of plasma proteins and cumulative metabolic effects of citrate anticoagulation.

### 3.2. Pathogenesis of Protein Depletion

#### 3.2.1. Immunoglobulin Depletion

Immunoglobulin G (IgG), the predominant antibody class in human plasma (normal range 800–1700 mg/dL), is maintained through synthesis by long-lived plasma cells and FcRn-mediated recycling that rescues IgG from lysosomal degradation. A single plasmapheresis session removing 750 mL of plasma extracts approximately 7–11 g of IgG, representing 5–10% of the total IgG pool [15]. At donation frequencies of once or twice per week, cumulative removal substantially exceeds the compensatory capacity of hepatic and lymphoid synthesis. The landmark SIPLA multicentre study of 3783 donors showed that IgG concentrations fell significantly with increasing donation frequency, with a 12.5% deferral rate attributable to low IgG values [16]. Cross-sectional surveys of high-frequency commercial plasmapheresis programmes document IgG below 700 mg/dL—the lower limit of the normal reference range—in 5–15% of donors completing more than 40 sessions annually [14]. IgM and IgA, which lack FcRn recycling and have shorter half-lives (approximately 5 and 6 days respectively), may decline more precipitously, though IgG depletion carries the predominant functional consequence for antimicrobial defence.

#### 3.2.2. Albumin and Coagulation Factor Depletion

Albumin—the most abundant plasma protein at 35–50 g/L—is synthesised exclusively in hepatocytes at approximately 10–15 g per day. Each plasmapheresis session removes 20–35 g, imposing a substantial synthetic demand. Although the liver can upregulate albumin production in response to reduced oncotic pressure, this response is constrained by nutritional protein adequacy and limited in donors with borderline hepatic reserve [17]. Longitudinal studies show progressive albumin decline in high-frequency donors, particularly those in the lowest tertile of dietary protein intake. Coagulation factors V, VII, VIII, X, XI, and fibrinogen are similarly removed with each session; modern apheresis protocols using saline or albumin replacement do not restore them, producing transient prolongation of prothrombin time and activated partial thromboplastin time in twice-weekly donors and potential cumulative haemostatic vulnerability [14].

### 3.3. Citrate-Mediated Metabolic Disturbance

All automated plasmapheresis procedures employ citrate as the extracorporeal anticoagulant. Citrate chelates ionised calcium and magnesium, and its accumulation constitutes a pathophysiological mechanism distinct from protein depletion. Acute citrate-induced hypocalcaemia—manifesting as perioral paraesthesia, carpopedal spasm, nausea, and, in severe cases, laryngospasm and cardiac arrhythmia—affects an estimated 1–5% of sessions to a clinically actionable degree, with subclinical ionised hypocalcaemia at higher frequency [18]. In chronic, high-frequency donors, repeated citrate metabolism to bicarbonate may produce cumulative metabolic alkalosis and suppress parathyroid hormone secretion, with some studies reporting reduced bone mineral density after more than 100 lifetime sessions, though prospective long-term data remain limited [19].

### 3.4. Incidence and Epidemiology

A German multicentre study found that donors completing more than 40 annual sessions showed significantly greater decrements in total protein and albumin than less frequent donors, with statistically meaningful declines emerging at 20 sessions per year in those with lower dietary protein intake [17]. A retrospective analysis of a large US apheresis programme found that donors donating for more than five consecutive years at high frequency had serum protein concentrations approximately 8–12% below age- and sex-matched controls [14]. A prospective Austrian cohort study found that donors with IgG below 700 mg/dL had a significantly higher rate of upper respiratory tract infections over 12 months compared to those with normal IgG [15]. Citrate-related adverse events collectively represent the most frequently reported class of donation-related event in hemovigilance data from plasmapheresis programmes globally [18].

### 3.5. Clinical Presentation

Early protein depletion is typically asymptomatic, detectable only through laboratory screening—underscoring the inadequacy of symptom-based monitoring. As immunoglobulin depletion deepens, susceptibility to recurrent and prolonged upper respiratory infections, sinusitis, and otitis media increases, resembling, in milder form, the clinical syndrome of secondary hypogammaglobulinemia from other causes. Albumin depletion may manifest as dependent pitting oedema, fatigue, and reduced physical reserve—features readily misattributed in the absence of systematic protein assessment. Acute citrate toxicity manifests with the characteristic tetany spectrum; chronic citrate-related effects may include non-specific fatigue, musculoskeletal symptoms, and restless legs syndrome.

### 3.6. Donor Protection Strategies

A multicomponent donor protection framework for regular plasmapheresis donors encompasses several evidence-supported elements. Protein-based eligibility screening—measurement of total serum protein and albumin at baseline and at minimum six-monthly intervals in donors completing more than 24 sessions per year, with the European Directorate for the Quality of Medicines recommending deferral when total protein falls below 60 g/L or albumin below 35 g/L—enables detection before clinical consequences supervene. Extension of quantitative IgG measurement to high-frequency donors substantially improves screening sensitivity. Frequency and volume limitation—adherence to WHO recommendations of no more than 25–30 donations per year for non-remunerated donors—is the single most structurally effective intervention; individualisation of intervals based on protein recovery kinetics represents the next refinement [20]. Citrate management protocols include prophylactic oral calcium supplementation at session commencement, slowing of return rates during symptomatic hypocalcaemia, and low-citrate apheresis platform settings; nutritional guidance should specify a protein intake of at least 1.0–1.2 g/kg body weight per day, with attention to calcium and vitamin D adequacy in donors with evidence of chronic citrate exposure [19]. Establishment of prospective donor health registries with systematic protein profiling and patient-reported outcome measurement is a public health research priority.

## 4. Acquired Absolute Lymphopenia as a Chronic Complication of Regular Platelet Donation by Apheresis

### 4.1. Pathogenesis of Apheresis-Related Lymphocyte Depletion

Platelet donation by apheresis—plateletpheresis—processes 3000–5000 mL of whole blood per session through a centrifugal extracorporeal circuit, retaining the platelet-rich fraction while reinfusing erythrocytes and plasma. The fundamental mechanism by which this procedure depletes lymphocytes is the non-selective retention of lymphocytes within the buffy coat generated during centrifugal separation: lymphocytes, monocytes, and platelets co-localise by virtue of their similar densities within the separation gradient, and their physical proximity to the platelet collection interface results in significant lymphocyte co-collection [21]. Quantitative studies document lymphocyte losses per session ranging from 0.6 × 10^9^ to 3.0 × 10^9^ cells, with a mean of approximately 1.0–1.5 × 10^9^ per donation. This co-collection is not a correctable calibration error but an inherent consequence of overlapping lymphocyte-platelet density ranges.

Importantly, the depletion is not uniformly distributed across lymphocyte subsets. Flow cytometric analyses of apheresis products and post-donation peripheral blood consistently show disproportionate losses of CD3^+^CD4^+^ T-helper lymphocytes and CD56^+^CD16^+^ natural killer (NK) cells, reflecting their buoyancy characteristics within the separation gradient [22]. The resulting distortion of the CD4^+^/CD8^+^ ratio—a clinical indicator of T-cell immune competence—has been documented in multiple cross-sectional cohorts of regular plateletpheresis donors. In donors using apheresis systems equipped with a leucocyte reduction system (LRS) chamber, lymphocyte losses are particularly pronounced; systems without this feature demonstrate substantially lower lymphocyte co-collection rates [23].

A further mechanistic contribution is activation-induced apoptosis: lymphocytes returned to the circulation after contact with the artificial extracorporeal circuit surfaces are subject to cytokine-mediated (IL-1β, IL-6, TNF-α) and Fas/FasL-mediated apoptosis in the hours following donation, constituting an additional route of lymphocyte attrition beyond direct collection. Recovery of lymphocyte count to baseline depends upon thymic output and peripheral homeostatic proliferation; in donors over 50 years—in whom thymic involution limits regeneration to peripheral clonal expansion—recovery between sessions is slower and potentially incomplete, rendering high-frequency donation in older donors particularly problematic [21].

### 4.2. Incidence and Epidemiology

Prevalence of lymphopenia (absolute lymphocyte count below 1.0 × 10^9^/L) attributable to regular plateletpheresis has been documented at 8–28% in donors completing more than 12 sessions per year, compared to 1–3% in age- and sex-matched non-donors, across studies from multiple countries [21,22]. The landmark Gansner et al. study at Brigham and Women’s Hospital, Boston, documented CD4^+^ T-cell counts below 200 cells/µL—a threshold of clinical significance in established secondary immunodeficiency—in 30% of the highest-frequency Trima Accel (LRS-equipped) platform donors, while no such depletion was found in Fenwal Amicus (non-LRS) donors [22,23]. The BEST collaborative study confirmed these findings across multiple international centres, documenting CD4^+^ below 200 cells/µL in 9.9% of frequent Trima donors versus 4.4% of Amicus donors versus 0% of whole blood donor controls [24]. A Swedish nationwide cohort study (SCANDAT3-S) of 74,408 apheresis donors demonstrated a dose-dependent increased risk of immunosuppression-related infections with increasing LRS plateletpheresis donation frequency [25].

Subset-specific depletion of NK cells is among the earliest detectable haematological abnormalities, with NK counts below normal reference range in 15–30% of donors completing more than 15 sessions annually [21]. Importantly, a follow-up study demonstrated that CD4^+^ lymphopenia persists in former frequent donors more than 12 months after cessation of plateletpheresis, demonstrating that recovery is slow and incomplete [26]. Preliminary unpublished data from our service show that CD4^+^ depletion below local normal limit of 460 cells/uL affects around 3% of the platelet donor pool independent of frequency of donation and none was below 200 cells/uL (personal communication). Formally, these donors should be deferred for an unknown length of time until reconstitution of the T-cell numbers. This might pose a supply difficulty, since T-cells have notoriously very long elimination half-lives over years. No data are available on the expected rate of return to donation in such cases; donors still need to be promptly replaced.

### 4.3. Clinical Presentation

In most affected donors, lymphopenia remains asymptomatic and detectable only through laboratory monitoring—rendering standard donation screening, which does not routinely include differential leucocyte counting, structurally incapable of identifying the complication. As depletion advances, the clinical picture resembles, in mild form, the secondary immunodeficiency syndromes seen in other causes of acquired lymphopenia: recurrent and prolonged viral upper respiratory infections, reactivation of latent herpesviruses (herpes zoster, oral herpes simplex, cytomegalovirus reactivation in the most severely depleted), and impaired clearance of opportunistic pathogens [25]. Of particular concern is the potential for sustained NK cell depletion to impair tumour immune surveillance, a risk that remains theoretically coherent but is not yet established by controlled malignancy incidence studies—an important evidence gap. The functional consequence of CD4^+^ depletion for vaccine responsiveness has been specifically studied in the context of COVID-19 vaccination, with studies demonstrating unaffected humoral responses in lymphopenic plateletpheresis donors [27]. Overall, evidence on the clinical significance of this kind of lymphopenia is considered contradictory concerning a harmful effect, because long-term prospective observations are lacking. Although in some studies recurrent viral infections were associated with the severity of lymphopenia [25], in others no differences in seroresponsiveness after vaccinations were noticed [24,27]. This might mean that T cell biological responsiveness might be intact at tissue level, despite numerical reductions in the peripheral blood.

### 4.4. Donor Protection Strategies

In view of the anticipated established risk for adverse health outcomes in persons with lymphopenia in the general population with absolute lymphocyte counts below 1.1 × 10^9^/L, it is intriguing to consider specific protection strategies for platelet donors exposed to this risk, although prospective evidence favouring morbidity in platelet donor cohorts is lacking. The most immediately implementable protection measure is incorporation of full differential leucocyte counting into the pre-donation eligibility screen, with absolute lymphocyte count below 1.0 × 10^9^/L, and CD4^+^ count below 400 cells/µL where flow cytometry is available, prompting temporary deferral and clinical review [21,24]. Longitudinal monitoring at six-monthly intervals in donors completing more than 12 sessions annually enables early identification of declining trajectories. Evidence supports restricting maximum annual sessions to 12–15 (from the 24–26 permitted in many jurisdictions), with further restriction to 8–10 for donors over 50 years; tailoring of intervals based on serial lymphocyte counts represents the logical, individualised refinement. Technological optimisation—specifically, preferential procurement of apheresis platforms without LRS chambers, or implementation of plasma rinseback protocols that partially restore lymphocytes to the donor—can substantially reduce lymphocyte loss without compromising platelet yield [23,24]. Donors with persistent CD4^+^ counts below 200 cells/µL should be indefinitely deferred and referred to clinical immunology. Hemovigilance registries should be expanded to systematically capture lymphocyte count trajectories, and large prospective cohort studies are urgently needed to quantify the clinical outcomes—infectious morbidity, malignancy incidence, vaccine responsiveness, and quality of life—attributable to donation-related lymphopenia.

## 5. Clonal Haematopoiesis of Indeterminate Potential in Regular Blood Donors

### 5.1. General Concepts

Clonal haematopoiesis of indeterminate potential (CHIP) refers to the presence of somatic mutations in haematopoietic stem or progenitor cells that drive clonal expansion in the absence of cytopenias or overt haematological abnormalities. Most CHIP-associated mutations occur in genes involved in epigenetic regulation—particularly DNMT3A, TET2, and ASXL1, which together account for approximately 70% of cases—with additional mutations in signal transduction, RNA splicing, and DNA damage response genes occurring less frequently [28]. CHIP is conventionally defined by a variant allele fraction (VAF) of at least 2%, with larger VAFs indicating greater clonal dominance and conferring higher risk of haematological malignancy and cardiovascular disease [29]. Age is the dominant determinant of prevalence: CHIP is rare below age 40, rises steeply with age, and affects more than 10–20% of individuals over 70 years [30].

In blood donors, the bone marrow is subjected to repetitive cycles of blood loss and haematopoietic recovery, mediated primarily by erythropoietin (EPO)-stimulated erythropoiesis. This proliferative stress may influence the expansion dynamics of certain clonal populations, potentially shaping a clonal architecture distinct from that seen in non-donor populations [31].

### 5.2. Evidence in Blood Donors

#### 5.2.1. Risk of Haematological Malignancy

Epidemiological evidence does not support an increased risk of haematological malignancy attributable to regular blood donation. A Swedish nationwide cohort study including more than one million donors and nearly 20 million person-years of follow-up found no increase in the risk of any haematological malignancy in regular donors; indeed, a modestly reduced risk of acute myeloid leukaemia (AML; standardised incidence ratio (SIR) 0.85, 95% CI 0.77–0.83) was observed alongside a modest excess of chronic lymphocytic leukaemia (CLL; SIR 1.07, 95% CI 1.01–1.15), with the latter showing no association with donation intensity in complementary nested case–control analysis [32]. The apparent AML risk reduction is biologically intriguing and, as discussed below, may reflect the predominance of low-risk EPO-responsive clonal variants in the donor population.

#### 5.2.2. Clonal Landscape in Frequent Donors

The most comprehensive molecular characterisation of clonal haematopoiesis in blood donors was recently published by Karpova et al. [31], who compared frequent donors (more than 100 lifetime donations) with sporadic donors (fewer than 10 donations) using deep targeted sequencing. Frequent donors showed comparable overall CHIP prevalence to age-matched sporadic donors, but with a distinct mutational spectrum: frequent donors were enriched for DNMT3A W305* variants, which preferentially expand under EPO stimulation and preserve normal erythroid differentiation, while high-risk premalignant DNMT3A R882 mutations—which expand under interferon-γ-mediated inflammatory conditions and exhibit impaired differentiation—were not enriched [31]. Functional experiments confirmed that W305*-bearing cells expand in EPO-stimulated conditions while contributing adaptively to erythrocyte regeneration. These findings indicate that frequent blood donation does not increase the overall burden of clonal haematopoiesis but does shape its character, favouring biologically low-risk, physiologically adaptive variants.

#### 5.2.3. Cardiovascular Outcomes

CHIP has been consistently linked to elevated cardiovascular risk in the general population through the pro-inflammatory activity of DNMT3A- and TET2-mutant myeloid cells, which promote vascular inflammation and accelerate atherosclerosis [33,34]. In blood donors, the cardiovascular impact of CHIP has not yet been systematically evaluated in dedicated longitudinal studies—a significant evidence gap. The predominance of TET2 variants as the second most common mutation type in blood donors [31] underscores the biological plausibility of vascular risk, even if current evidence does not establish a direct clinical link in the donation context.

### 5.3. Biological Plausibility: Clonal Competition and EPO-Driven Selection

The concept of clonal competition is central to understanding CHIP dynamics in blood donors. The selective pressures imposed by the bone marrow microenvironment—EPO-driven regenerative stress versus inflammatory cytokine-mediated stress—determine which clonal populations expand preferentially [35]. In regular blood donors, EPO-stimulated erythropoiesis creates a microenvironment that favours DNMT3A variants capable of efficient erythroid differentiation, while suppressing the inflammatory milieu that would otherwise promote high-risk R882 variants. This may provide a biological basis for the modest AML risk reduction observed epidemiologically, suggesting that the selective clonal environment created by regular donation may paradoxically be protective against transformation of the highest-risk DNMT3A clones—a tentative “healthy donor microenvironment” hypothesis warranting formal investigation, as a hypothesis-generating observation.

### 5.4. Clinical Implications and Policy Considerations

From a clinical standpoint, the dominant determinants of CHIP-associated risk—age, clone size (VAF), and mutation type—apply equally to donors as to the general population. Current evidence does not support routine CHIP screening in blood donors, given uncertain clinical actionability and the predominance of low-risk variants in this population [36]. However, as sequencing technologies become affordable and widely deployed, incidental CHIP detection is expected to increase; blood services should develop evidence-based frameworks for counselling donors with incidentally identified CHIP, particularly older individuals or those with extended donation histories [37]. Whether donor-enriched DNMT3A variants remain stable or undergo gradual evolution over decades of continued donation is not yet known—a critical unanswered question for long-term donor safety. The question of transfusion transmission of CHIP-associated clones, while not documented for blood transfusion, warrants vigilance given case reports of clonal engraftment in haematopoietic stem cell transplantation under permissive conditions [38].

## 6. Vitamin and Micronutrient Deficiencies in Regular Blood Donors

### 6.1. Pathogenesis and Scope

While iron deficiency dominates the micronutrient deficiency literature in blood donors, accumulating evidence indicates that regular donation imposes measurable losses of a range of vitamins and trace elements, with the potential for clinically significant deficiency in donors with pre-existing dietary insufficiency. Whole blood donation removes plasma-soluble vitamins and intracellular micronutrients proportional to the donated volume; apheresis modalities impose additional modality-specific losses. The regulatory focus on haemoglobin and, increasingly, ferritin screening has meant that vitamin status has received comparatively little systematic attention in donor management frameworks.

### 6.2. B-Vitamins: Folate, B12, and B6

Folate (vitamin B9) and cobalamin (vitamin B12) are both essential for nucleotide synthesis, erythropoiesis, and neural function. Each whole blood donation removes approximately 25–50 µg of folate and small quantities of B12, proportional to their plasma and erythrocyte concentrations. While individual donation-related losses are modest relative to total body stores, cumulative losses in high-frequency donors—particularly those with dietary insufficiency or increased metabolic demand—may contribute to suboptimal status over time [20,39,40]. In countries with mandatory folate food fortification, clinically overt deficiency from donation alone is uncommon; however, donors following unfortified plant-based diets are at risk of both folate and B12 depletion, the latter because B12 is present almost exclusively in animal-source foods and its absorption is dependent upon intrinsic factor [41,42]. Elevated homocysteine—a sensitive marker of combined folate, B12, and B6 insufficiency—has been documented in some cohorts of regular blood donors, with potential implications for vascular risk [42,43].

Pyridoxine (vitamin B6) participates in amino acid metabolism, haem biosynthesis, and neurotransmitter production. Its plasma concentration is modestly reduced in regular donors in some studies, a finding of uncertain clinical significance in isolation but potentially relevant in donors who are simultaneously iron-depleted and micronutrientmarginal. The interaction between B-vitamin status and haematopoietic regeneration after donation is mechanistically plausible: folate and B12 deficiency impairs DNA synthesis in rapidly dividing erythroid progenitors, potentially limiting the rate of haemoglobin recovery between donations and contributing to macrocytic anaemia superimposed on iron deficiency in nutritionally vulnerable donors [20,39,40,44,45].

### 6.3. Vitamin D

Vitamin D—a fat-soluble secosteroid with roles in calcium and phosphate homeostasis, immune regulation, and cardiovascular biology—is removed in small quantities with each whole blood donation via its plasma-bound and erythrocyte-associated fractions. The primary clinical concern regarding vitamin D in blood donors, however, relates to the indirect effects of chronic citrate exposure in plasma and platelet apheresis donors: citrate-mediated hypocalcaemia stimulates secondary hyperparathyroidism, which drives 1α-hydroxylase activity and accelerates conversion of 25-hydroxyvitamin D to the active 1,25-dihydroxyvitamin D, potentially depleting 25-hydroxyvitamin D stores [19]. Additionally, the immune-modulatory effects of vitamin D are relevant in the context of donation-related lymphopenia, as vitamin D deficiency independently impairs T-cell function and NK cell activity. Observational studies report a higher prevalence of vitamin D insufficiency (25-hydroxyvitamin D below 50 nmol/L) in regular platelet and plasma apheresis donors compared to non-donors in some cohorts, though causality has not been formally established [44,46,47]. It should be acknowledged here that causality is difficult to establish because prevalence of Vit D deficiency is already high by 20–50% in otherwise healthy subjects in the general population [48].

### 6.4. Vitamin A, Vitamin E, and Zinc

Fat-soluble vitamins A and E are removed with each donation in quantities proportional to their plasma concentrations; cumulative losses across frequent donations may affect antioxidant defence capacity and immune function in donors with marginal dietary intake, though clinical deficiency from donation alone has not been demonstrated in well-nourished populations. Zinc, an essential trace element for immune function, DNA repair, and erythropoiesis, is present in both plasma and erythrocytes; whole blood donation removes approximately 0.3–0.5 mg of zinc per session. In donors who donate frequently and consume diets low in bioavailable zinc—particularly those with high phytate intake—cumulative zinc losses might theoretically contribute to suboptimal immune function and impaired wound healing, though systematic data from blood donor cohorts remain limited [39].

### 6.5. Vitamin B2 (Riboflavin) and Pathogen Reduction

Riboflavin (vitamin B2) merits specific mention in the context of apheresis donation in blood services employing riboflavin-based pathogen reduction technology (Mirasol, Terumo BCT) [49,50]. This technology uses riboflavin in conjunction with ultraviolet light to inactivate pathogens in platelet and plasma products; residual riboflavin in the apheresis product is retained in the component, not returned to the donor, but the ultraviolet irradiation process may alter riboflavin metabolites and potentially influence the donor’s systemic riboflavin balance if implemented as part of a routine donation programme. While systematic donor riboflavin monitoring in this context has not been formally evaluated, the potential for measurable riboflavin depletion in high-frequency donors warrants investigation.

### 6.6. Donor Protection Strategies

A pragmatic, risk-stratified approach to micronutrient monitoring in blood donors should identify those at highest risk: premenopausal women, donors following plant-based diets, donors completing more than 20 donations annually, elderly donors, and donors with symptoms suggestive of micronutrient depletion. For these groups, measurement of serum vitamin B12, folate, 25-hydroxyvitamin D, and zinc at baseline and annually might be reasonable, although suggestive evidence does not support this unconditionally. Structured dietary counselling addressing not only iron but the full spectrum of micronutrients depleted by donation should be standard practice, with specific guidance on B12 supplementation for plant-based donors and vitamin D supplementation in those with insufficient solar exposure or dietary intake. Blood services with national fortification policies for folate and other vitamins should ensure these policies extend effective coverage to their donor populations. Research into the cumulative micronutrient impact of apheresis donation—particularly in the context of pathogen reduction technology—represents an important knowledge gap.

## 7. Psychological Aspects of Chronic Blood Donation

One of the best-documented long-term psychological complications of regular blood donation is conditioned needle- and procedure-related anxiety following a vasovagal reaction (VVR). VVRs affect roughly one to seven percent of donations and, rather than fading with repeated exposure, can become sensitised in vulnerable donors: each episode strengthens an aversive association between the donation setting and the physical symptoms experienced, producing escalating anticipatory anxiety, needle phobia, and a generalised fear of medical procedures [51]. This conditioned avoidance is the strongest single predictor of donors discontinuing their donation career, reducing return probability by more than half. In donors who sustain injury during a syncopal episode, there is a theoretical, though not yet formally studied, pathway to post-traumatic symptoms. Unlike transient mood changes around a single donation, this represents a genuinely chronic, self-reinforcing psychological sequela with direct behavioural consequences for donor retention [52].

The most systematic prospective study, which followed regular whole blood donors over eight weeks across seven psychological dimensions (energy, mood, anxiety, somatic complaints, etc.), found that in experienced donors (≥3 prior donations), donation is typically followed by a transient positive mood elevation and increased energy—consistent with the “warm glow” model—and that this positive effect generally outlasts the negative somatic effects (fatigue, light-headedness). Importantly, the study found no evidence of chronic, sustained deterioration in any psychological dimension attributable to donation itself over the follow-up period [52].

On the stress side, the stress response some donors show is more about the emotional novelty of a never-before-experienced situation than the donation act itself. Donors who avoid major adverse events show habituation (declining stress with repetition); donors who experience adverse events (especially vasovagal reactions) instead show sensitisation—escalating anticipatory anxiety rather than habituation.

## 8. Integrated Discussion: The Case for Multiparameter Donor Health Monitoring

The six complication domains reviewed in this article share structural features that carry important collective implications for donor management policy. In each domain, the pathophysiology is mechanistically coherent and biologically well-characterised; the epidemiology, while heterogeneous across studies, is directionally consistent; and the clinical consequences—spanning from asymptomatic laboratory perturbation to measurable functional impairment—exist on a spectrum whose upper end represents genuine donor harm. In each domain, current eligibility screening is inadequate to detect the complication at a stage where intervention is most effective: haemoglobin screening misses iron depletion; clinical inquiry misses lymphopenia and protein depletion; standard donation records cannot capture CHIP dynamics or micronutrient trends.

A convergent finding across all six domains is the critical importance of donation frequency and cumulative donation burden as the principal modifiable risk determinants. Iron deficiency risk rises steeply with donation frequency in whole blood donors; protein and immunoglobulin depletion in plasmapheresis donors is strongly correlated with annual session count; lymphopenia in plateletpheresis donors is most severe in those with the highest lifetime donation totals; CHIP dynamics in frequent versus sporadic donors differ in ways that reflect the cumulative haematopoietic proliferative burden; and micronutrient deficiency risk is amplified by the compounding of multiple donation-related losses across years or decades. The implication is that frequency and interval regulation—not simply minimum eligibility criteria—is a primary lever for donor health protection.

A second shared theme is the inadequacy of population-level, one-size-fits-all management in the face of substantial inter-individual variability in risk. Premenopausal women and plant-based dietary adherents are disproportionately vulnerable to iron and micronutrient depletion. Older donors face impaired lymphopoietic recovery after plateletpheresis. Donors with pre-existing marginal protein synthesis capacity are more vulnerable to plasmapheresis-related protein depletion. These gradients of risk argue for personalised, longitudinally guided donor management—the “precision haemovigilance” model analogous to ferritin-guided interval adjustment for whole blood donors, generalised to encompass the full spectrum of biologically relevant parameters.

The ethical dimensions of donor health in this context are substantial. Voluntary, non-remunerated blood donors contribute an irreplaceable social good, and blood services owe them not only the negative duty of avoiding harm but the positive duty of active monitoring and protection. The fact that complications are often subclinical in early stages—and therefore not volunteered by donors—places the detection obligation firmly on the blood service. Transparent communication of laboratory findings to donors, alongside evidence-based counselling, is both an expression of this ethical obligation and, in practice, a powerful tool for donor retention: donors who receive clear, accurate information about their health status and are supported in managing donation-related risks are more likely to remain committed long-term donors than those whose concerns are dismissed. The financial issue of this monitoring strategy is also worth of discussion. In most cases it burdens the budget of the Blood Service, since health insurances mostly do not cover preventive services. As an example in Switzerland a systematic investigation of lymphocyte subpopulations, IgG, IgM, IgA concentrations and total protein content would cost approximately an additional EUR 140.- for laboratory testing of one platelet donor by apheresis (standard pricing according to the official Swiss List of Laboratory Analyses). Although this might considerably differ in other European countries, it still poses a financial challenge, considering the magnitude of platelet donor pools in the communities.

### Towards a Comprehensive Donor Health Monitoring Framework

**Figure 1:**
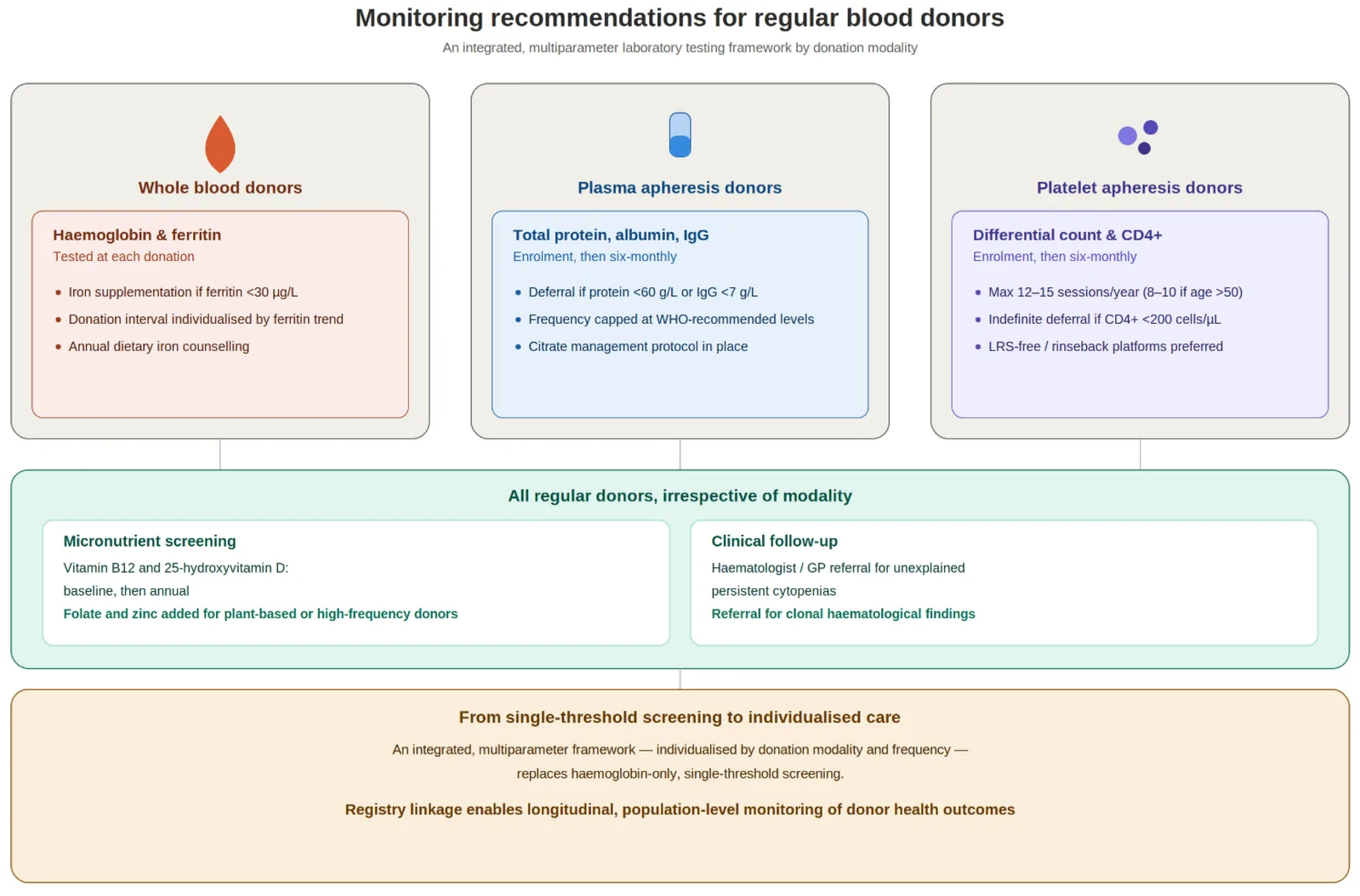
Tentative monitoring recommendations for regular blood donors within the context of a donor protection strategy (extracted from the above proposed Section “Towards a Comprehensive Donor Health Monitoring Framework”).

Drawing on the evidence reviewed across all five complication domains, a tentative integrated multiparameter monitoring framework for regular donors can be proposed (Figure 1, Table 1). For all regular whole blood donors: haemoglobin and ferritin at each donation; dietary iron counselling at enrolment and annually; oral iron supplementation offered post-donation where ferritin is below 30 µg/L; inter-donation intervals individualised by ferritin trajectory. For regular plasma apheresis donors: total protein, albumin, and quantitative IgG at enrolment and six-monthly; frequency cap at WHO-recommended levels; citrate management protocol and nutritional protein counselling; deferral for total protein below 60 g/L or IgG below 7 g/L. For regular platelet apheresis donors: full differential leucocyte count including absolute lymphocyte count and CD4^+^ count (flow cytometry in high-frequency donors) at enrolment and six-monthly; maximum 12–15 sessions annually; 8–10 sessions for donors over 50 years; technological preference for LRS-free or rinseback-equipped platforms; indefinite deferral with clinical immunology referral for CD4^+^ below 200 cells/µL. For all regular donors: baseline and annual assessment of vitamin B12, 25-hydroxyvitamin D, and, in plant-based or high-frequency donors, folate and zinc; haematologist or general practitioner referral for unexplained persistent cytopenias or clonal haematological findings. Registry linkage to enable longitudinal health outcome monitoring at population level.

## 9. Conclusions and Future Research Priorities

Regular blood donation, across all modalities, imposes a spectrum of acquired health consequences on the donor that is more extensive, and more clinically significant, than has historically been acknowledged within regulatory and medical frameworks oriented primarily around recipient safety. Iron deficiency—common, well-characterised, and preventable—represents the paradigmatic case: a condition whose pathophysiology is fully understood, whose epidemiology is robustly documented, and whose management tools are evidence-based and available, yet which remains underdetected and undertreated because the structural constraints of current eligibility screening prioritise haemoglobin over the more informative ferritin measure. The complications of plasma donation, platelet donation, and the emerging molecular consequences for haematopoietic clonal dynamics illustrate that iron deficiency, for all its prevalence, is not the only consideration—and that a narrow focus on the most prevalent complication risks leaving a broader landscape of donor vulnerability unaddressed.

Future research priorities to close critical evidence gaps and eliminate current uncertainties include: large-scale, prospective, long-duration cohort studies designed to quantify the clinical outcomes—infectious morbidity, malignancy incidence, cardiovascular events, cognitive function, vaccine responsiveness, and quality of life—attributable to each complication domain; randomised trials comparing different monitoring and management strategies (ferritin-guided intervals, IgG-based deferral policies, supplementation regimens) in diverse donor populations; mechanistic studies of the long-term evolution of donor-enriched CHIP clones and their cardiovascular and haematological consequences; investigation of the cumulative micronutrient impact of apheresis donation, particularly in populations with dietary vulnerability; and development and validation of predictive models identifying donors at highest risk across multiple complication dimensions, to guide targeted and proportionate monitoring intensity.

The sustainability of voluntary blood donation systems—systems that are not simply logistical conveniences but the expression of a profound social compact between donors and the healthcare systems they support—depends upon demonstrable evidence that the health of donors is taken as seriously as the safety of the blood supply they provide. The evidence reviewed in this article provides both the scientific foundation and the ethical imperative for a substantive upgrade in the ambition, comprehensiveness, and individualisation of donor health monitoring.

## Figures and Tables

**Table 1:** Tentative laboratory testing schedule as monitoring recommendations for regular blood donors (extracted from the above proposed integrated multiparameter monitoring framework, Section 8).

Laboratory Test	Interval	Action/Threshold
**Whole blood donors**		
Hemoglobin	Each donation	—
Ferritin	Each donation	Oral iron supplementation offered if <30 µg/L; donation interval individualised by ferritin trajectory
**Plasma apheresis donors**		
Total protein	Enrolment, then six-monthly	Deferral if <60 g/L
Albumin	Enrolment, then six-monthly	—
Quantitative IgG	Enrolment, then six-monthly	Deferral if <7 g/L
**Platelet apheresis donors**		
Full differential leucocyte count (incl. absolute lymphocyte count)	Enrolment, then six-monthly	—
CD4^+^ count (flow cytometry in high-frequency donors)	Enrolment, then six-monthly	Indefinite deferral + clinical immunology referral if <200 cells/µL
**All regular donors**		
Vitamin B12	Baseline, then annual	—
25-hydroxyvitamin D	Baseline, then annual	—
Folate	Baseline, then annual (plant-based or high-frequency donors)	—
Zinc	Baseline, then annual (plant-based or high-frequency donors)	—
Clinical follow-up	As needed	Hematologist/GP referral for unexplained persistent cytopenias or clonal hematological findings

Dark green and light green colors help distinguish headings.

## Data Availability

No new datasets were generated or analysed in this narrative review. All information discussed is derived from previously published literature.
